# Experimental station Bernina at SwissFEL: condensed matter physics on femtosecond time scales investigated by X-ray diffraction and spectroscopic methods^1^

**DOI:** 10.1107/S160057751900331X

**Published:** 2019-04-15

**Authors:** Gerhard Ingold, Rafael Abela, Christopher Arrell, Paul Beaud, Pirmin Böhler, Marco Cammarata, Yunpei Deng, Christian Erny, Vincent Esposito, Uwe Flechsig, Rolf Follath, Christoph Hauri, Steven Johnson, Pavle Juranic, Giulia Fulvia Mancini, Roman Mankowsky, Aldo Mozzanica, Roland Alex Oggenfuss, Bruce D. Patterson, Luc Patthey, Bill Pedrini, Jochen Rittmann, Leonardo Sala, Matteo Savoini, Cristian Svetina, Thierry Zamofing, Serhane Zerdane, Henrik Till Lemke

**Affiliations:** a SwissFEL, Paul Scherrer Institut, 5232 Villigen PSI, Switzerland; b Institut de Physique de Rennes, Université de Rennes, 35042 Rennes CEDEX, France; c Institute for Quantum Electronics, Eidgenössische Technische Hochschule (ETH) Zürich, CH-8093 Zurich, Switzerland; d Ecole Polytechnique Fédérale de Lausanne, CH-1015 Lausanne, Switzerland

**Keywords:** FEL, X-ray, pump–probe, time-resolved

## Abstract

The Bernina instrument at the SwissFEL Aramis hard X-ray free-electron laser is designed for studying ultrafast phenomena in condensed matter and material science. Ultrashort pulses from an optical laser system covering a large wavelength range can be used to generate specific non-equilibrium states, whose subsequent temporal evolution can be probed by selective X-ray scattering techniques in the range 2–12 keV. For that purpose, the X-ray beamline is equipped with optical elements which tailor the X-ray beam size and energy, as well as with pulse-to-pulse diagnostics that monitor the X-ray pulse intensity, position, as well as its spectral and temporal properties. The experiments can be performed using multiple interchangeable endstations differing in specialization, diffractometer and X-ray analyser configuration and load capacity for specialized sample environment. After testing the instrument in a series of pilot experiments in 2018, regular user operation begins in 2019.

## Abstract 2

The Bernina instrument at SwissFEL Aramis employs laser-pump and X-ray-probe techniques to selectively excite and probe the electronic, magnetic and structural dynamics in condensed matter systems on the femtosecond time scale and under extreme conditions.

## 1. Introduction   

Hard X-ray free-electron lasers (XFELs) (Emma *et al.*, 2010 ▸; Ishikawa *et al.*, 2012 ▸; Kang *et al.*, 2017 ▸) provide ultrashort X-ray pulses which combine the sensitivity to molecular and crystal structures, as well as to core electron levels, with sufficient time resolution to resolve electronic transitions and atomic motions. Over the last decades, high-flux and high-repetition-rate synchrotron X-ray sources have revolutionized the insights into static electronic and nuclear structures of condensed matter materials, by combining elastic and inelastic diffraction methods with spectroscopic sensitivity to specific electronic and magnetic states of selected elements in a material (Kim *et al.*, 2011 ▸; Gibbs *et al.*, 1988 ▸; Murakami *et al.*, 1998*a* ▸,*b* ▸). Such methods have revealed material structures in bulk and surface, of nanometre-sized objects and crystals, as well as the complicated interplay of electronic, magnetic and nuclear structure in strongly correlated electron systems. The femtosecond (10^−15^ s) time resolution provided at XFELs opens experimental access to short-lived out-of-equilibrium states which determine transitions between charge states, magnetic and electronic phases and atomic/crystalline structures (Murakami *et al.*, 1998*b* ▸; Trigo *et al.*, 2013 ▸; Beaud *et al.*, 2014 ▸; Clark *et al.*, 2013 ▸; Singer *et al.*, 2016 ▸). The results indicate that additional insight into complex material functions can be gained in the time domain. Materials with complex inter­actions of charge-transfer energy, magnetic exchange, Jahn–Teller splitting, hopping integral or Hubbard interaction (∼0.15–4 eV) exhibit interesting phenomena like ferro- and antiferromagnetism, metal-to-insulator transitions, multiferroics, charge and spin density waves (CDW, SDW), colossal magnetoresistance (CMR), high-*T*_c_ superconductivity or topological insulation. When perturbing the equilibrium by external parameters while following the involved degrees of freedom in the time domain, their coupling and relevance for the phenomenon can be studied in much greater detail. FELs represent here an important complementary tool as they allow sensitive X-ray methods to be applied in the femtosecond time domain, like resonant and non-resonant X-ray diffraction (tr-RXRD) of elastic and inelastic scattering (tr-RIXS) which are selective to electronic, magnetic and nuclear structure.

The SwissFEL Aramis XFEL (Milne *et al.*, 2017 ▸) provides X-ray pulses in the energy range from 1.77 to 12.4 keV (Table 1 ▸), which covers the *K*-edges of elements from P to As (*Z* = 15 to 33), the *L*-edges of most 4*d* and 5*d* materials (Rb to Au) as well as the *M*-edges from Dy (*Z* = 66) onward. The pulse length theoretical shorter limit down to 0.2 fs bears the potential to even resolve ultrafast electronic transitions, in combination with subsequent slower degrees of freedom like atomic motions. These qualities have motivated the design of the Bernina instrument, specialized for studying coupled phenomena in condensed matter by (*a*) selective manipulation of specific transitions or degrees of freedom and (*b*) selective probing of the ensuing electronic, magnetic and structural dynamics. We describe here the design and realization of the original concept (Ingold & Beaud, 2013 ▸), as well as the status of commissioning and pilot experiments over a ten-month frame shared with accelerator and Aramis commissioning, installations and developments.

## 2. Instrument design   

### 2.1. X-ray source   

The linear accelerator of SwissFEL consists of an S-band photoinjector linac and three C-band booster linacs that increase the electron energy to 2.1 GeV, 3.0 GeV and 5.8 GeV, respectively. The nominal normalized slice emittance after the injector is 0.2 µm for a bunch charge of 200 pC (Schietinger *et al.*, 2016 ▸). A two-stage magnetic bunch compression after the injector (BC1) and the first linac (BC2) is used to reach a nominal peak current of 3 kA. The linac can simultaneously drive two XFELs operated in self-amplified spontaneous emission (SASE) mode at 100 Hz, the hard X-ray Aramis undulator (0.1–0.7 nm) at 5.8 GeV and the soft X-ray Athos undulator (0.65–5 nm) at 3.0 GeV (currently under construction). SwissFEL can be tuned from high charge mode (200 pC) to low charge mode (10 pC), providing between 50 and 5 fs (FWHM) short pulses, respectively. Aramis uses a segmented planar, variable-gap short-period undulator (15 mm, *K* = 0.1–1.8) to generate horizontally linear polarized light (Calvi *et al.*, 2018 ▸), at 0.1% bandwidth in normal SASE mode (‘pink beam’; presently achieved average bandwidth: 0.4%, Fig. 4). A four-magnet electron energy collimator before the undulators facilitates two special modes, namely the large-bandwidth mode (relative bandwidth ∼4%) (Hernandez *et al.*, 2016 ▸) and an attosecond mode which exploits full compression of a 10 pC pulse which is expected to produce sub-femtosecond short pulses. Self-seeding at ångstrom wavelengths with increased spectral brightness with respect to SASE is foreseen for the the future (Amann *et al.*, 2012 ▸; Prat & Reiche, 2013 ▸). Electron bunch arrival-time monitors (BAMs) with electro-optical detection scheme (Angelovski *et al.*, 2015 ▸) are installed after each bunch compressor and at the end of the undulator. They provide non-destructive, shot-to-shot arrival-time information relative to a highly stable pulsed optical reference with resolution better than 5 fs and less than 10 fs drift per day (Arsov *et al.*, 2014 ▸). In addition, two C-band transverse deflecting structures are installed at the end of the third linac measuring the longitudinal charge profile and the arrival time of the compressed bunches in two-bunch mode with a resolution of a few femtoseconds.

### 2.2. X-ray optics and diagnostics   

The FEL pulses delivered by the Aramis undulator are generally diagnosed non-destructively for intensity and position by a high-transmission ionization chamber with split electrodes. This gas monitor performs over an energy range of 0.025–12 keV at an intensity and position relative (absolute) accuracy of 1% (10%) and about 10 µm, respectively. A solid state attenuator (silicon and diamond filters) allows the FEL intensity to be reduced. A single-pulse X-ray spectrometer can be inserted in the range 4–12 keV (Rehanek *et al.*, 2017 ▸). It uses a thin diamond transmission grating that splits off the first diffraction order in the horizontal plane to illuminate a bent Si crystal spectrometer. The resolving power Δ*E*/*E* is 10^−5^–10^−4^ in a simultaneously accessible measurement window of 0.5% bandwidth.

**Figure 1:**
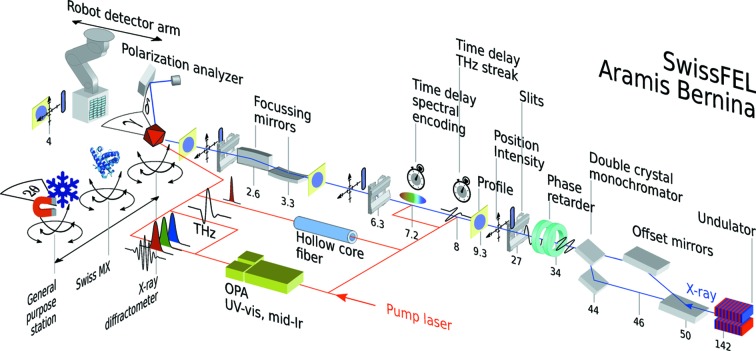
Schematic layout of the Aramis Bernina pump/probe instrument including selected elements. The pump laser source can be converted to different energies and pulse lengths for exciting a specimen. Part of it is used to measure the time delay with respect to the X-ray pulses from the Aramis FEL. The X-ray beamline consists of a set of monochromator crystals as well as beam steering and focusing optics. The X-ray pulse spatial profile, position and intensity are measured at multiple positions in the beamline. The experiments can be carried out at multiple endstations which can be translated into the focused FEL beam.

The Aramis optical beamline design enables fast (<1 min) switching of the FEL beam between the three experimental stations Alvra, Bernina and Cristallina (Follath *et al.*, 2016 ▸). The Bernina beamline propagates straight in the horizontal direction from the Aramis undulator trajectory (Fig. 1 ▸). In the vertical direction, the beam path to the experimental station is separated by 20 mm for radiation safety in a dedicated X-ray optics hutch. A vertical deflecting periscope geometry for offset mirrors and monochromator matches the beam paths of the full SASE bandwidth and monochromatic beam, respectively. A pair of bendable plane elliptical offset mirrors (OMs) deflect the beam vertically by 6 mrad. Adjustment of curvature allows modest prefocusing in the vertical plane depending on the beam divergence at a given photon wavelength. To prevent single-shot damage by the intense FEL beam and to enhance the reflectance and the critical angle over the full Aramis photon energy range (1.8–12.4 keV), the mirrors are coated with a low-*Z* (10 nm B_4_C on top of 36 nm SiC) and mid-*Z* (15 nm B_4_C on top of 20 nm Mo) bilayer leaving a blank area of uncoated silicon between them. Uncoated silicon has the lowest critical energy of about 10 keV and may be used at low energies when harmonic rejection above 3.5 keV becomes important. Switching from pink to monochromatic mode is accomplished by OM retraction and insertion of the double-crystal monochromator (DCM). The DCM has three crystal pairs mounted on common Bragg-rotation axes of incidence angular range 5–70°. Si(111) and Si(311) crystals can be used for standard and high-resolution applications (calibration example in Fig. 4). An additional pair of InSb(111) crystals extends the photon energy range to tender X-rays down to 1.8 keV. The DCM geometry with translation perpendicular to the second crystal surface enables an adjustable beam offset up to 32 mm. An installation of additional harmonic rejection mirrors, foreseen in the original design, has received lower priority, based on first measurements in the tender X-ray regime where the focusing mirrors (see below) sufficiently rejected the higher harmonics.

Behind the DCM, a diamond phase retarder is foreseen in order to control the X-ray polarization. The option to create and control circular polarization will increase the sensitivity and selectivity in resonant X-ray diffraction (RXRD) to magnetic and oriented electronic structures. The rotation of the linear polarization plane also facilitates studying oriented structures at identical pump/probe geometry in time-resolved resonant X-ray diffraction (tr-RXRD). Operated in Bragg or Laue transmission geometry a polarization of 80–95% has been achieved above 7 keV at synchrotron beamlines (Strempfer *et al.*, 2013 ▸) and at XFELs (Suzuki *et al.*, 2014 ▸). A double-stage phase retarder with two crystals operated in series allows to compensate for depolarizing effects due to angular divergence and energy spread (Scagnoli *et al.*, 2009 ▸; Francoual *et al.*, 2013 ▸) and also achieve higher degrees of linear polarization. Undulator-based polarization control (Schmidt & Calvi, 2018 ▸) in an ‘afterburner’ configuration (Lutman *et al.*, 2016 ▸) is investigated for future upgrade.

Before transportation of the FEL beam from the optics hutch to the Bernina experimental station, the X-ray beam trajectory, profile and intensity can be measured and aligned by multiple beam-defining apertures (four-blade slits), profile monitors (based on Ce:YAG scintillators) and quadrant detection intensity and position monitors (Tono *et al.*, 2011 ▸). They allow well defined conditions to be reproduced before and after deflection from X-ray optics.

Pulse-to-pulse arrival-time jitter between the X-ray pulses and the optical laser may severely limit the temporal resolution available for time-resolved experiments. Different methods are currently commissioned that measure the relative laser pump and X-ray arrival time at different locations for every pulse. The separate arrival time of FEL electron bunches and pump laser pulses can be measured by the BAM at the end of the undulator line and a laser arrival monitor (LAM) with respect to a master reference clock at an expected overall time resolution of around 50 fs FWHM. Improved and more representative temporal resolution can be reached by direct cross-correlation of FEL X-rays and optical laser pulses within the beamline and near the experiment. This is achieved by a streaking of X-ray pulse generated photoelectrons by a THz electric field generated from the pump laser source (Gorgisyan *et al.*, 2017 ▸). Thanks to a gas target, the method can be used over the entire X-ray photon energy range at high X-ray transmission. An X-ray time resolution of ≤40 fs FWHM is expected. A relative cross-correlation resolution down to a few femtoseconds can be reached by the spectral encoding timing diagnostics (Hartmann *et al.*, 2014 ▸; Harmand *et al.*, 2013 ▸; Bionta *et al.*, 2011 ▸). As the performance in this case depends highly on sufficient X-ray intensity, a location close to the X-ray focus (1 m from sample location) can be used additionally to the built-in device indicated in Fig. 1 ▸.

**Figure 2:**
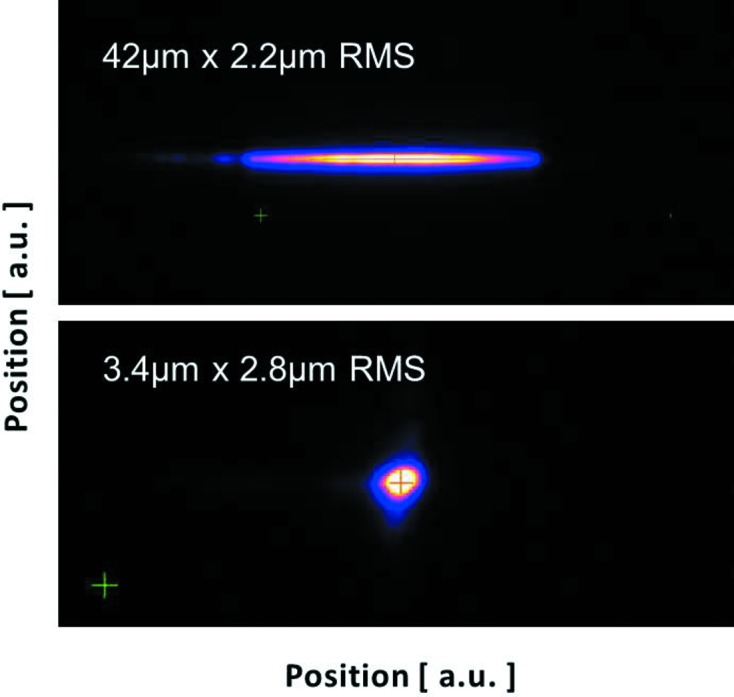
Focal spot for a line and point focus at 6.6 keV (third harmonic) measured at the Bernina sample position during early commissioning (fundamental FEL energy at 2.2 keV). The focal spot was measured in air with a profile monitor after transmission through a diamond (50 µm) window separating the beamline vacuum system from the experimental area.

A pair of focusing mirrors in orthogonal Kirkpatrick–Baez (KB) geometry are installed at 2.6 m working distance upstream of the sample position. They combine achromaticity with high transmission over a large energy range (Yumoto *et al.*, 2013 ▸). The mirrors (B_4_C/Mo-coating, 500 mm length) operate at grazing incidence angle, which can be chosen between 4 and 12 mrad total deflection. This allows suppression of higher harmonics from the SASE beam by a factor of 10^−3^ to 10^−4^. With mirror benders and a profile error of ≤2 nm in the mirror centre, the spot at the sample location can be adjusted between 2 and ∼300 µm FWHM, independently in the horizontal and vertical directions (Fig. 2 ▸).

In order to control and reproduce the beam pointing of the KB-focused FEL beam, additional profile and intensity/position monitors are installed upstream and downstream with respect to the sample position. They are mounted on high-precision motorized tables, a six degrees of freedom heavy-load hexapod holding the upstream diagnostics and a two-axis translation table supporting the downstream diagnostic devices. Those allow the diagnostics to be positioned at identical positions relative to the FEL beam, also upon change of the KB grazing angle. The upstream diagnostic chamber houses an optional arrival-time monitor, an X-ray profile and intensity monitor, as well two slit systems to remove beam halo.

### 2.3. Timing synchronization and optical laser   

A global timing reference for all timing-sensitive components at SwissFEL is generated by an optical master oscillator. For optimized overall phase noise performance it is synchronized to an RF master oscillator which itself is locked to a 10 MHz rubidium frequency standard. The integrated absolute jitter from 10 Hz to 1 MHz is as low as 50 fs FWHM (Milne *et al.*, 2017 ▸). The most critical clients of SwissFEL require <35 fs FWHM jitter and down to 10 fs peak-to-peak temporal drift per day (Schietinger *et al.*, 2016 ▸). Critical clients such as the experimental laser system, the BAM and LAM monitors, are fibre-coupled to pulsed optical links.

The laser system (Erny & Hauri, 2016 ▸) for the experimental stations consists of two identical commercial 100 Hz Ti:sapphire amplifier systems with a compressed output energy of 20 mJ at 800 nm. These lasers are installed above the experimental stations in a separate clean room with air (±0.1°C) and humidity (40–45%) control. To ensure simultaneous and independent operation of the experimental stations both lasers can serve either station. The uncompressed amplifier output is sent through an evacuated transfer line to optical tables in the Bernina hutch where it is split into two branches, one for the experiment and the other for timing diagnostics. The diagnostic branch is used to operate the THz arrival-time monitor for which the THz electrical field is generated using the tilted pulse front scheme in LiNbO_3_ (Hebling *et al.*, 2002 ▸).

The experimental branch includes the LAM to measure the laser arrival near the experiment and non-linear conversion stages offering a broad range of pump frequencies. The LAM is based on a spectrally resolved cross-correlation with a resolution of 12 fs FWHM (Csatari Divall *et al.*, 2015 ▸). For experimental use, a range of parameters can be derived from the compressed output of 10 mJ, <30 fs, 800 nm (Table 2 ▸). A short-pulse option offers <10 fs pulses with >200 µJ at 800 nm generated by spectral broadening via self-phase modulation in a gas-filled hollow core fibre and subsequent pulse compression (Nisoli *et al.*, 1996 ▸). Frequency tuning is achieved via an optical parametric amplifier (OPA), combined with subsequent frequency mixing stages covering a broad wavelength range from the UV up to the mid-IR (0.24–15 µm). The signal branch of the OPA output can also be used to generate intense single-cycle THz pulses by optical rectification of near-IR pulses in organic non-linear crystals (DAST, OH1, DSTMS) (Hauri *et al.*, 2011 ▸; Ruchert *et al.*, 2013 ▸; Kozina *et al.*, 2017*b* ▸) or by the tilted pulse front technique in LiNbO_3_ (Hebling *et al.*, 2002 ▸; Kozina *et al.*, 2017*a* ▸), yielding field strengths up to a few hundreds of kV cm^−1^ in the frequency range 0.3–3 THz.

Single-cycle THz pulses can be used to excite (resonantly or non-resonantly) large-amplitude atomic motions with electric and magnetic fields exceeding 1 MV cm^−1^ and 1 T, respectively. Single-cycle THz pulses are intrinsically stable with respect to their carrier-envelope phase (CEP) and the optical cycle is long compared with the FEL pulse duration, allowing the field-induced dynamics to be tracked. So far mostly resonant field driven excitations in the 0.3–2 THz range probed by FEL X-ray diffraction have been applied to study soft mode dynamics in the prototypical ferrolectrics Sn_2_P_2_S_6_ (Grübel *et al.*, 2016 ▸), BaTiO_3_ (Chen *et al.*, 2016 ▸) and SrTiO_3_ (Kozina *et al.*, 2017*a* ▸,*b* ▸), the magneto-electric excitation of the spin cycloid in the multiferroic TbMnO_3_ (Kubacka *et al.*, 2014 ▸) and the initiation of the metal-to-insulator transition in VO_2_ (Gray *et al.*, 2018 ▸).

By exploiting selective excitation and control of higher-energy modes in the frequency range 15–20 THz (20–15 µm), recent experiments have demonstrated efficient non-linear coupling from the resonantly excited IR-active mode to Raman modes along the symmetry breaking coordinate (Först *et al.*, 2011*a* ▸). The suppression of magnetic and orbital order (Först *et al.*, 2011*b* ▸, 2015 ▸), enhanced superconductivity in high-*T*_c_ superconductors (Mankowsky *et al.*, 2014 ▸; Mitrano *et al.*, 2016 ▸; Fausti *et al.*, 2011 ▸) or ultrafast switching of the ferroelectric polarization (Mankowsky *et al.*, 2017 ▸; von Hoegen *et al.*, 2018 ▸) has been demonstrated. In one case non-linear electron–phonon coupling was found to dominate directly the charge order melting in a doped manganite (Esposito *et al.*, 2017 ▸).

With the potential to reach sub-10 fs time resolution, field-induced dynamics at mid-IR frequency can be studied with CEP-stable narrow-bandwidth and frequency-tunable pulses. For that purpose a CEP-stable system based on two independent OPAs (TOPAS-twins, Light Conversion) is being installed at Bernina. Developments that extend the available tuning range to the so-called THz gap in the range 4–20 THz (15–75 µm) are closely followed for potential future implementation (Liu *et al.*, 2017 ▸; Cartella *et al.*, 2017 ▸).

The large range of laser parameters available for experiments at Bernina requires flexible incoupling with respect to the X-ray beamline. Especially for mid-IR/THz wavelengths, flexible mounting provisions for optical support tables and breadboards are provided close to the sample location in order to facilitate use of small-focal-length focusing optics. For UV/VIS and IR wavelengths, exchangeable dedicated in-coupling vacuum chambers for high vacuum and intermediate/helium atmosphere are mounted on the heavy-load upstream diagnostics hexapod table between the diagnostics element and the experimental endstations. They are separated by an X-ray window (50 µm CVD diamond) from the diagnostics chamber which is retractable for windowless operation.

### 2.4. Endstations   

**Figure 3:**
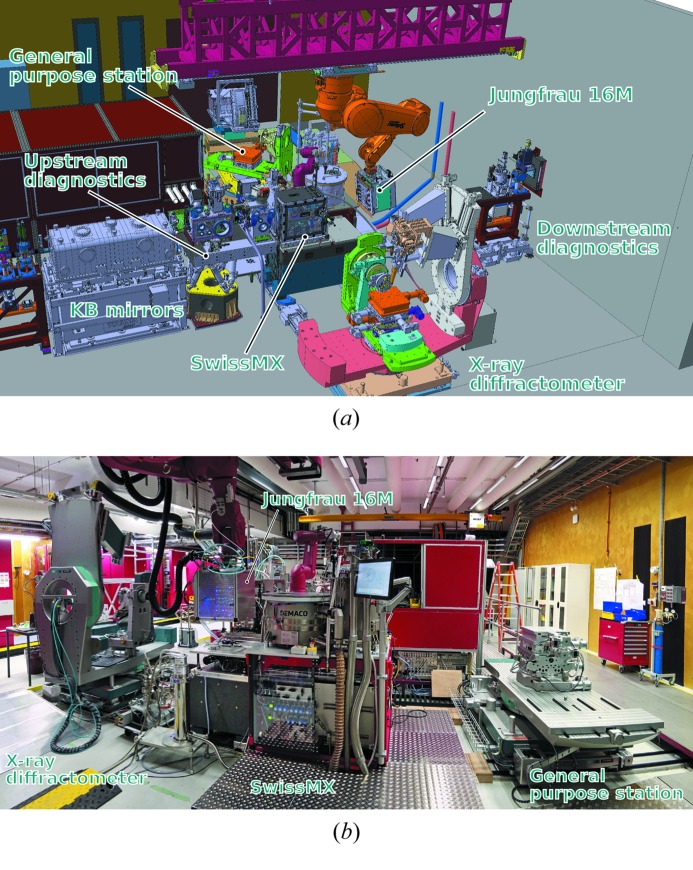
Experimental area and endstations. Top: CAD-view showing the robot detector mount and the three experimental endstations. Bottom: photograph in the direction upstream of X-ray propagation.

The endstation design (Ingold *et al.*, 2016 ▸) emphasizes rapid reconfiguration capability in terms of sample environment, spectrometers and detector geometries to support a wide variety of experiments. For this purpose two endstations for X-ray pump–probe (XPP) experiments are installed, a general purpose station (Bernina-GPS) as well as a six-circle X-ray diffractometer (Bernina-XRD). Both endstations are mounted on a high-precision rail system oriented perpendicular to the X-ray trajectory, allowing them to be exchanged and reproducibly positioned at the sample location (Fig. 1 ▸). Heavy-load capability allows the stations to support and precisely align a variety of custom sample stage modules such as goniometers and sample environmental setups which can be swapped and mounted on both Bernina-GPS and Bernina-XRD. Sample modules provided by an experimental team or user group may include cryogenic vacuum chambers, cold-finger cryostats, superconducting high-field magnets or cryogenically cooled pulsed magnet systems and high-pressure diamond anvil cells. As a third station which is mounted temporarily for periods of time on the same rail system, a fixed-target protein crystallography station (SwissMX) is operated at Bernina (Fig. 3 ▸).

The Bernina-GPS station uses a high-precision positioning platform that can be aligned with respect to the FEL beam trajectory in three degrees of freedom compensating for pointing changes induced by the KB focusing system. This platform supports two concentric, vertical rotation axis circle stages for sample environment and detector systems. The sample circle can be combined with sample diffractometer modules; the detector circle can support heavier detectors or analyzers (≤300 kg) at about 1 m distance from the sample interaction.

The Bernina-XRD station platform is identical to the Bernina-GPS station except for the detector circle configuration. The detector arm has two rotation circles, with a vertical and a horizontal rotation axis. It supports two motorized translation detector stages carrying a 1.5M Jungfrau area detector and a vacuum polarization analyzer stage with angular collimation and point detector, respectively (Strempfer *et al.*, 2013 ▸). The stages are offset by 20° to allow rapid switching between the analyzer stage and the Jungfrau detector. The sample–detector distance of 790 mm and pixel size of 75 µm × 75 µm provides an angular resolution of 0.00547° (Δ*Q* = 5.4 × 10^−4^ at 11.2 keV).

On the single sample circles of both endstations an additional choice of three pre-configured sample diffractometer stages can be mounted. They support different motion degrees of freedom and ranges for sample environment of different size and weight. A κ-goniometer with ascending motorized horizontal sample axis, κ axis and additional six degrees of freedom is used to position sample systems of up to ∼0.3 kg weight. This leaves space above the sample and on the free side so that, by suitable combination of rotations, a wide range of reciprocal space can be accessed without parts of the diffractometer obstructing either the incident or scattered X-ray beams or the optical pump. For intermediate sample environments like small vacuum chambers, a hexapod goniometer consisting of a hexapod mounted on a horizontal rotation axis can be used. For sample environments like large sample chambers, cryostats or superconducting magnets, a non-magnetic heavy-load five-degrees-of-freedom goniometer (load capacity of 700 kg) can be used on both endstations.

The detection stages for both endstations can be combined with a ceiling-mounted robot detector arm, which normally holds a large-area Jungfrau 16M pixel detector. The sample–detector distance can be varied along the beam axis by a translation stage holding the entire robot arm, for example, to perform SAXS measurements (Kim *et al.*, 2017 ▸) with momentum resolution Δ*Q* = 1.2 × 10^−4^ Å^−1^ (10 keV) at a maximum distance of 3.1 m. It can further be used for coherent Bragg diffraction on nano- and micro-crystalline materials (Clark *et al.*, 2013 ▸), time-domain measurements of density fluctuations by diffuse scattering (Trigo *et al.*, 2013 ▸), and combined X-ray emission (XES) and diffraction experiments to resolve the electronic and geometric structure of catalytic and macromolecular micrometre-sized crystals (Kern *et al.*, 2013 ▸).

The Bernina-XRD endstation is intended particularly for sample environments at extreme temperatures, pressures or magnetic fields which facilitates different classes of experiments: suppression of stripe order (Fausti *et al.*, 2011 ▸) or enhancement of superconductiviy far above *T*_c_ (Hu *et al.*, 2014 ▸; Mankowsky *et al.*, 2014 ▸) in high-*T*_c_ materials, the competition of CDW and superconducting phases at low temperatures (*T* ≤ 10 K) and high magnetic fields (*H* ≥ 30 T) (Jang *et al.*, 2016 ▸; Chang *et al.*, 2012 ▸, 2016 ▸; Gerber *et al.*, 2015 ▸), or pressure (*P* ≤ 20 GPa) driven quantum critical behaviour in 2D magnetic systems (Jaramillo *et al.*, 2009 ▸; Haravifard *et al.*, 2012 ▸; Feng *et al.*, 2012 ▸), and iron-based superconductivity (Takahashi *et al.*, 2015 ▸).

The heavy-load detector arm of Bernina-GPS allows experiments with large emission spectrometers as used for resonant inelastic X-ray scattering (RIXS), which has been demonstrated as time- and momentum-resolved hard X-ray RIXS (tr-qRIXS) at an XFEL (Dean *et al.*, 2016 ▸). A compact five-analyzer RIXS spectrometer (*R* = 1 m) (Moretti Sala *et al.*, 2018 ▸) will be available at Bernina-GPS to study the dynamics of charge and *dd*-excitations in 3*d* transition metal oxides (*K*-edge XES/RIXS), and dispersive spin-exciton and magnon excitations in 5*d* systems (*L*-edge RIXS) that exhibit strong spin–orbit coupling (SOC). For measurements at a well defined momentum in *q*-space only the central analyzer will be used. Spherical bent diced analyzers (Gog *et al.*, 2013 ▸) are operated on a Rowland circle such that the sample, central analyzer and detector are all mounted on a circle of radius *R*/2 (Johan geometry). The X-rays backscattered from the analyzer are detected with a 250 K 2D Jungfrau detector with pixel size of 225 µm (h) × 25 µm (v). The overall resolution at 11.2 keV (Ir *L*_3_-edge) is 50–70 meV for an Si(444) DCM and Si(844) diced-crystal analyzer. Adopting a horizontal scattering plane and dispersion in the vertical plane complies well with an asymmetric X-ray footprint (vertical focus) on the sample to preserve energy- and time-resolution when matching of the penetration depth of the laser-pump and X-ray-probe is required at grazing incidence θ ≃ 0.5–5° (Johnson *et al.*, 2008 ▸, 2010 ▸). Tr-qRIXS requires high spectral flux and would greatly benefit from self-seeding operation (Amann *et al.*, 2012 ▸) of Aramis in the future to enhance the spectral brightness by a factor of 5–8.

The Bernina-XRD station is intended to enable experiments that require precise reciprocal space measurements. Besides time-resolved resonant and non-resonant XRD, it additionally allows measurements of polarization-resolved tr-RXRD. Time-resolved diffraction experiments on correlated systems such as mixed-valence CMR maganites (Beaud *et al.*, 2009 ▸, 2014 ▸) can be extended to selectively probe different components of the resonant scattering tensor by measuring the polarization dependence in the presence of cooperative oriental alignment caused by competition between charge- or orbital-ordered phases and magnetic phases. This is done by resolving the scattering into the respective σ–σ′ and σ–π′ polarization channels where σ (π) and σ′ (π′) denote the polarization perpendicular (parallel) to the scattering plane for the X-rays in the entrance and exit channel, respectively (Zimmermann *et al.*, 2001 ▸; Grenier *et al.*, 2004 ▸). From the intensities of the σ- and π-components the Poincaré–Stokes parameters *P*_1_ and *P*_2_ for the linear polarization state are derived and the degree of circular polarization *P*_3_ is inferred from 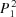 + 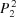 + 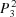 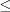 1 (Paolasini *et al.*, 2007 ▸; Mazzoli *et al.*, 2007 ▸).

In magnetic systems the transient magnetic order due to 4*f*–5*d* magnetic coupling (Rettig *et al.*, 2016 ▸) or frustrated exchange interactions (Johnson *et al.*, 2012 ▸, 2015 ▸) could be resolved. Similarly the magnetic and orbital ordering in a bilayer perovskite ruthenate (Bohnenbuck *et al.*, 2008 ▸) and the magnetic structure in a bilayer SOC iridate (Kim *et al.*, 2012 ▸) have been determined. In a combined RXRD and RIXS study the unresolved magnetic ground state in a prototypical pyrochlore iridate has been clarified (Donnerer *et al.*, 2016 ▸). In some cases polarization analyzer scans are combined with scans of the azimuthal angle where the sample is rotated around the scattering vector. These combined scans directly measure the symmetries of the tensors involved in resonant scattering amplitudes. However, pump–probe experiments impose geometric constraints and azimuthal scans will be of limited use in practice. Instead the use of diamond phase retarders in combination with a polarization analyzer would be the preferred solution for polarization-resolved tr-RXRD measurements.

Fixed-target serial femtosecond crystallography (FT-FX) (Hirata *et al.*, 2014 ▸; Feld *et al.*, 2015 ▸; Roedig *et al.*, 2015 ▸, 2016 ▸; Opara *et al.*, 2017 ▸) is an additional activity at Bernina. The SwissMX endstation (Pedrini *et al.*, 2017 ▸) is installed at Bernina from time to time for FT-FX experiments. The main element of the station is a sample diffractometer placed inside an experimental chamber suitable for various data collection schemes which require precise positioning of the protein samples when hit by the femtosecond X-ray pulses. The station also includes a cryo-jet, room-temperature sample reservoirs, a liquid-nitrogen sample reservoir for up to 500 sample pins, as well as a dedicated robot for automated sample exchange. Both serial scanning data collection (FT-SFX) at 100 Hz from 3D micro-crystals of size <5 µm positioned on a chip as well as synchrotron-like data collection of larger crystals by rotation are foreseen. The measurements can be performed in air or in helium atmosphere with X-rays as low as 5 keV. During data collection the samples are maintained either at room temperature or at cryogenic temperatures using a nitrogen or helium gas stream cryocooler. The diffraction images from the protein crystals will be collected with the 16M Jungfrau detector mounted on the robot detector arm. By positioning the detector immediately downstream of the experimental chamber a resolution of better than 1 Å can be achieved with 12 keV radiation.

### 2.5. Detector and data acquisition   

The Jungfrau pixel detector has specifically been developed for the needs of the Aramis endstations (Mozzanica *et al.*, 2014 ▸, 2018 ▸) by the detector group at PSI. It is a charge integrating detector using a hybrid architecture consisting of silicon sensors bump-bonded with a readout ASIC (application specific integrating circuit). The standard Jungfrau ASIC consists of 256 × 256 pixels, each pixel being 75 µm × 75 µm in size. Each pixel incorporates a preamplifier with three gains, a switching block that enables an automatic gain switching pixel by pixel, a sampling stage consisting of a storage array for 16 images, and a readout buffer. The output of the ASIC is a signal proportional to the number of photons interacting with the Si sensor. Arrays of 2 × 4 chips (512 × 1024 pixels) form a module of about 0.5 Mpixel. The readout board uses a FPGA to generate control signals for the readout ASICs and receives the data stream from the ADCs (14 bit) which digitize the multiplexed analogue output from the chips. The readout time set by the ADC clock speed (40 MHz) corresponds to a frame rate of 2.5 kHz.

**Figure 4:**
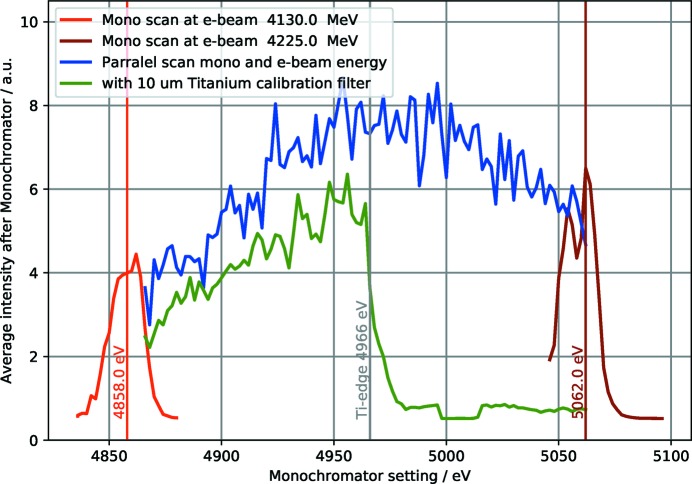
SwissFEL Aramis average bandwidth measurement (for two different electron energy settings). The monochromator energy can be adjusted in parallel to the electron energy in order to scan a larger energy range, *e.g.* for calibration purpose.

Experiments at Bernina will profit from several features of the Jungfrau detector. Firstly, the noise level, well below Poisson statistical fluctuations across the full dynamic range, is low enough at the highest gain to allow single-photon detection even at tender X-ray energies in the range 2–4 keV. Secondly, automatic gain switching with three levels of gain for individual pixels raises the overall dynamic range of the detector to 10^4^ per pixel per pulse. This is particulary important for simultaneous measurement of diffuse scattering or Bragg diffraction from charge-, orbital- or spin order together with up to 5000 times more intense Bragg reflections. Thirdly, the energy resolution capability (see Fig. 4 ▸) allows efficient discrimination of elastic from fluorescence signals at lower count rates, which can be used to, for example, suppress background signals. Special use-oriented variations of the sensor design have been developed, like the high spatial resolution in one dimension (pixel size 25 µm × 225 µm) for spectrometer applications. For in-vacuum operation the sensors and detector chips can be mounted in vacuum (10^−6^ mbar), cooled by a cooling plate (∼20 W per module) and separated by a vacuum flange from the readout board which is operated in air.

SwissFEL uses a de-centralized data acquisition system recording data asynchronous and synchronous to the individual FEL pulses. Asynchronous data are predominantly based on the channel access protocol EPICS. Synchronous data are based on ZeroMQ streams which are tagged by Id numbers for each FEL pulse (pulse-Ids) from their data source. This allows scalable operation of even multiple large data sources like the 16M Jungfrau detector, through separate individual data streams to disk storage and recombination of data from individual pulses by data buffering and pulse-Id based data access. Experimental data are stored to multiple data files (hdf5 format) by a system of separated data acquisition systems for experimental data from Jungfrau detectors, 2D camera images, scalar and 1D waveform data as well as asynchronous data. The systems match the individually saved data ranges by communicating the requested pulse-Ids and data time-stamps. 2D camera images, scalar and 1D waveform data and asynchronous data are in addition permanently buffered for diagnostics purposes over hours or days.

Combined manual and programmatic control of the various subsystems is possible by a high-level scripting framework of Python libraries. It can be used standalone or as a library for specialized applications. It includes standard data acquisition functionality like step-by-step and continuous scans, and can access acquired data for automatization of procedures. A distributed online data processing framework is under development.

### 2.6. Commissioning and first experiments   

The Aramis undulator has been in first operation since the end of 2016, and has been commissioned step by step within the following year with interruptions for installations.

**Figure 5:**
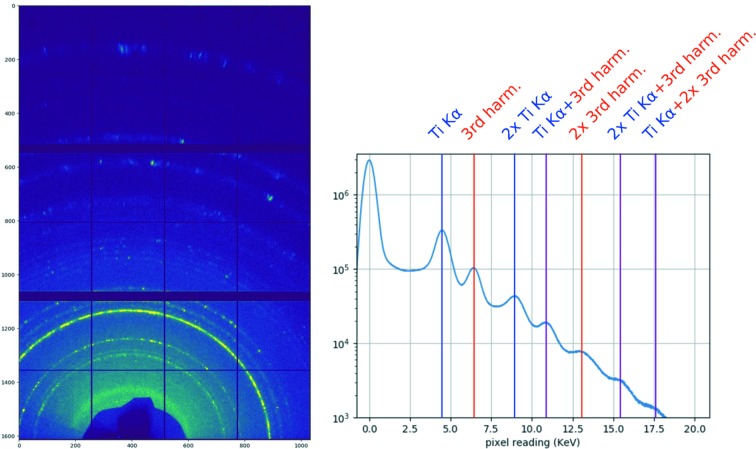
Jungfrau detector (1.5M, three modules) powder diffraction data from Ti_3_O_5_. Left: average pattern of 200 pulses recorded at 6.6 keV third harmonic using the Aramis pink beam at 2.2 keV fundamental SASE energy. The image is generated by femtosecond grazing-incidence powder diffraction of a Ti_3_O_5_ nanoparticle pellet. Right: histogram over all pixels in all 200 patterns averaged on the left. The digital data are calibrated to equivalents of single-photon energy. The peak at 0 keV shows the noise distribution of the unexposed pixels (‘zero photon peak’), very well separated from the lowest-energy photons measured at the Ti *K*α emission (4.5 keV). Those can, in turn, be well distinguished from the elastically scattered third harmonic photons at 6.6 keV, which allows the data to be filtered during analysis for, for example, the fluorescent background signal. Pixels which have been hit by multiple fluorescence and/or scattered photons can be distinguished as labelled in the histogram.

**Figure 6:**
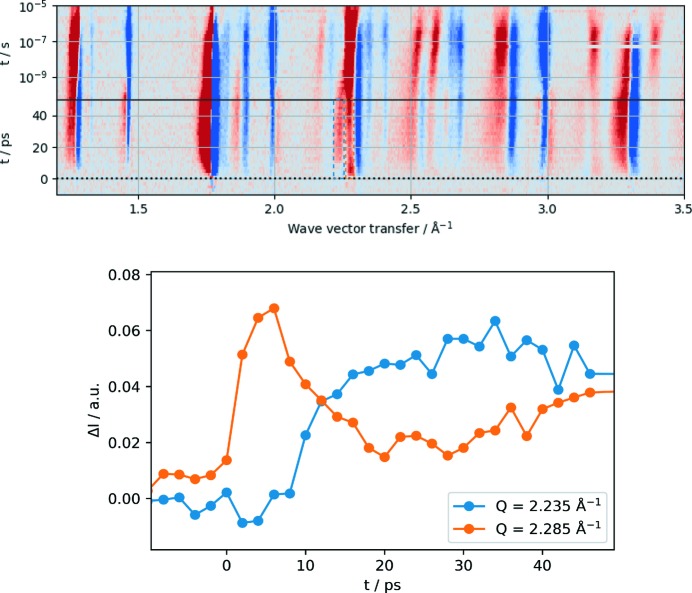
Bernina first pilot experiment. Top: light-induced differential scattering pattern collected from Ti_3_O_5_ nanocrystals in Debye–Scherrer geometry as a function of momentum transfer and time delay. The powder patterns have been collected with a 2D 1.5M Jungfrau pixel detector. Bottom: relative change of diffraction intensity as a function of time delay between the 800 nm pump and X-ray probe at 6.6 keV. For two momentum transfers indicated by the dashed box in the top panel a distinct different time behaviour is observed at shorter times. The instrumental time resolution derived from Bi(111) diffraction intensity prior to the experiment was about 350 fs FWHM.

The first pilot experiment at SwissFEL was performed in November 2017 at the Bernina instrument after several weeks of commissioning of prioritized elements in the X-ray beamline. A light-induced semiconductor-to-metal transition in titanium pentaoxide Ti_3_O_5_ was measured at Bernina by femtosecond grazing-incidence powder diffraction (M. Cammarata, Rennes University). The saturated SASE pulse energy was 200 µJ at 2.20 keV (*K* = 1.2) of the fundamental at electron energy 2.38 GeV, bunch charge 205 pC and repetition rate 10 Hz. The experiment was performed using pink X-ray beam at 6.6 keV and 0.1% bandwidth using the third harmonic of the fundamental. The instrumental time resolution of about 350 fs FWHM was measured by light (800 nm) induced changes in the diffracted Bi(111) intensity from a 40 nm bismuth expitaxial thin film of 40 nm (Johnson *et al.*, 2008 ▸). The time resolution was mainly limited by electron bunch compression and pump laser synchronization. No timing diagnostics for time jitter and drift corrections were applied. X-ray powder diffraction patterns from a polished nanoparticle pellet of 20–30 nm-sized crystallites were recorded with a 1.5M Jungfrau pixel detector. The energy resolution of the detector allowed gating to suppress background due to Ti *K*α fluorescent photons (Fig. 5 ▸). The pump fluence at 800 nm and spot size 300 µm × 300 µm FWHM was varied in the range 0.16–0.44 mJ. The focal spot size of the X-ray was 100 µm (h) × 5 µm (v) FWHM and the incidence angle was varied in the range 0.3–0.5° to probe the phase transition as a function of depth at the nanoscale. The time-resolved powder patterns shown in Fig. 6 ▸ hold information on the volume and crystalline phase fractions as a function of time. The metallic phase has a higher unit cell volume and thus the semiconductor-to-metal transition may occur only after pressure relaxation. To clarify this the time resolution of 350 fs FWHM is sufficient because volumetric changes of ∼25 nm-sized crystallites proceed on the acoustic timescale of ∼0.5 ps nm^−1^.

The second pilot experiment was performed in March 2018. Single-cycle THz pulses were used to directly drive lattice vibrations in the ferroelectric (FE) model compound Sn_2_P_2_S_6_ with a time-dependent electric field (M. Savoini and S. Johnson, ETH Zurich). Lowering the temperature below 337 K induces a soft-mode driven displacive phase transition to a ferroelectric state. The structural changes associated with the paraelectric-to-ferroelectric phase transition can mainly be attributed to a displacement of the Sn^2+^ ion and its direction roughly coincides with the direction of the polarization in the ferroelectric phase. The displacement can also directly be linked to a soft phonon mode near 1 THz at room temperature. By exciting this soft phonon mode with broadband THz pulses the structural modulation has been measured directly and quantitatively in a recent tr-XRD experiment (Grübel *et al.*, 2016 ▸) at the FEMTO slicing facility at the SLS (Beaud *et al.*, 2007 ▸; Ingold *et al.*, 2008 ▸). In a subsequent experiment at the X-ray pump–probe instrument (XPP) at the LCLS (Chollet *et al.*, 2015 ▸), additional modes by using higher THz fields have been observed. In continuation of these experiments the pilot experiment was designed to identify and quantify dissipation mechanisms in the strongly non-equilibrium state that possibly impact the feasibility of FE switching.

**Figure 7:**
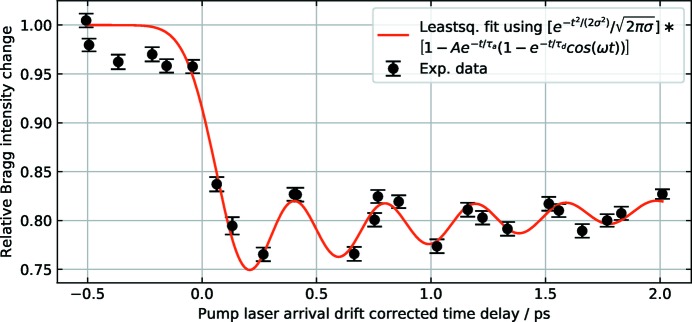
Jitter-limited time resolution. Laser (800 nm) induced coherent *A*_1*g*_ phonon mode in a 40 nm thin (111)-oriented film of crystalline bismuth at an incident fluence of 1 mJ cm^−2^. The fit by the model function in the plot legend reveals a time resolution of 110 fs σ (r.m.s.). Timing-drifts of the pump laser timing were corrected for by the laser arrival monitor (LAM).

**Figure 8:**
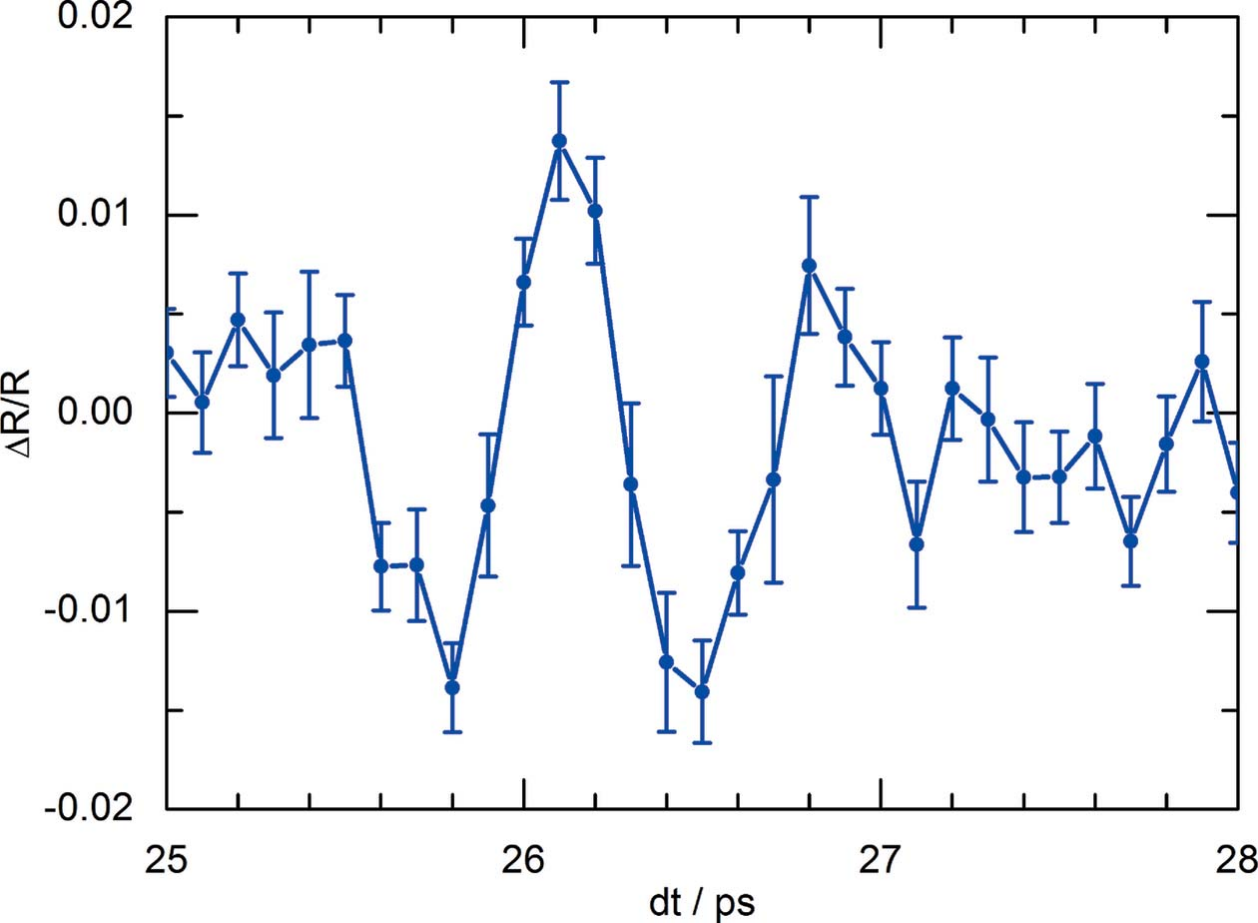
Time evolution of the normalized diffraction intensity of the (332) reflection in the ferroelectric Sn_2_P_2_S_6_ induced by a THz electric field of 350 kV cm^−1^. Data were acquired during the second Bernina pilot experiment.

The experiment was performed with monochromatic X-rays at 6.0 keV using the third harmonic at 0.015% bandwidth. Aramis was operated at 2.5 GeV and bunch charge 200 pC at 10 Hz to reach saturation at 2.0 keV (*K* = 1.45) of the fundamental. The transverse deflecting structures (TDS) installed prior to the second pilot experiment allowed good control of the bunch length by the two bunch compressors BC1 and BC2. The saturated pulse energy (pulse length) of the SASE pulses was 205 µJ (90 fs FWHM) and 350 µJ (170 fs FWHM) depending on compressor settings. The electron beam arrival-time jitter measured with TDS was about 70 fs and 90 fs FWHM, respectively. The shot-to-shot jitter measured independently with a BAM installed after BC1 was in agreement with the TDS measurements. The timing drift measured with the BAM was 100 fs peak-to-peak over 4 h. By measuring the laser (80 nm, 40 fs FWHM, 1 mJ cm^−2^) induced Bi(111) diffraction peak using the short bunch setting, the coherent *A*_1*g*_ phonon oscillation is clearly noticed as shown in Fig. 7 ▸. The X-ray beam was focused to <10 µm horizontally to minimize geometric time broadening. The instrumental time resolution is about 260 fs FWHM without applying jitter or drift corrections for the X-ray time arrival, but applying drift correction using the LAM arrival information of the pump laser pulses. The remaining jitter is mainly due to the synchronization of the pump laser which was accurate to 190 fs FWHM. In the meantime, the synchronization of the pump laser has been improved to 35 fs FWHM. Laser-induced pump–probe experiments with time resolution <200 fs FWHM can therefore be performed without the need for shot-to-shot timing corrections. This is due to the low timing jitter of the electron beam as also noticed at other XFEL facilities (Kang *et al.*, 2017 ▸). Broadband single-cycle THz pulses at centre frequency near 1 THz were generated through optical rectification of NIR pulses (2.4 mJ) at 1.3 µm in an OH1 organic crystal. The electrical field strength measured by electro-optic sampling was 350 kV cm^−1^. A two-step procedure was followed to ensure overlap of the X-ray and THz pulses on the sample (Grübel *et al.*, 2016 ▸). By using a collinear pump–probe geometry on a (010)-oriented Sn_2_P_2_S_6_ bulk crystal the structural dynamics of the soft phonon mode has been probed by the (332) and (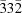) diffraction peaks via coplanar asymmetric diffraction. The incidence angle was 15° to limit the X-ray penetration depth. The measured response shown in Fig. 8 ▸ is in agreement with earlier measurements showing that THz-pumped tr-(R)XRD experiments can now be performed at Bernina.

## 3. Conclusions   

The SwissFEL Aramis Bernina instrument is designed as a versatile tool for femtosecond pump–probe X-ray experiments in condensed matter physics and material science to explore the coupling dynamics between atomic, electronic and magnetic constituents that lead to novel functional properties in materials. Emphasis is on developing selective excitation and probe techniques to stimulate and observe the response of the system when at the same time it is adiabatically tuned under extreme conditions. First pilot experiments have been performed to demonstrate IR and THz-pumped tr-XRD experiments with time resolution of about 260 fs FWHM, limited by the arrival-time jitter between the X-ray and laser beams. Instrumentation to accurately measure pulse-by-pulse the X-ray intensity and spectrum, as well as the relative X-ray arrival, is under commissioning in order to exploit the time-resolved potential of Aramis.

## Figures and Tables

**Table 1:** X-ray FEL parameters at the SwissFEL Bernina instrument

Undulator beamline	Aramis
Electron energy	Up to 5.8 GeV
Electron bunch charge	20–200 pC
Photon energy	1.77–12.4 keV
Pulse energy	1 mJ
Pulse duration	0.1–100 fs
Repetition rate	100 Hz

**Table 2:** Laser options at the SwissFEL Bernina instrument

Experimental branch	30 fs†, 10 mJ, 800 nm
Short pulse option	<10 fs, 300 µJ

HE-TOPAS‡ (pump: 10 mJ, 30 fs)	
Signal and idler	1160–2600 nm§
VIS-UV extensions	240–1160 nm§
DFG extensions	2600–15000 nm§

TOPAS twins‡ (pump: 10 mJ, 100 fs)	
DFG extensions	2600–20000 nm§ ¶

† Option 100 fs.

‡ For details see http://lightcon.com/Products/opa-topas.html.

§ Pulse energies and durations on best effort basis.

¶ CEP-stable.
